# Identification of a New Potential SARS-COV-2 RNA-Dependent RNA Polymerase Inhibitor via *Combining* Fragment-Based Drug Design, Docking, Molecular Dynamics, and MM-PBSA Calculations

**DOI:** 10.3389/fchem.2020.584894

**Published:** 2020-10-30

**Authors:** Mahmoud A. El Hassab, Aly A. Shoun, Sara T. Al-Rashood, Tarfah Al-Warhi, Wagdy M. Eldehna

**Affiliations:** ^1^Department of Pharmaceutical Chemistry, School of Pharmacy, Badr University in Cairo (BUC), Cairo, Egypt; ^2^Department of Microbiology and Immunology, Faculty of Pharmacy, Damanhour University, Damanhour, Egypt; ^3^Department of Pharmaceutical Chemistry, College of Pharmacy, King Saud University, Riyadh, Saudi Arabia; ^4^Department of Chemistry, College of Science, Princess Nourah Bint Abdulrahman University, Riyadh, Saudi Arabia; ^5^Department of Pharmaceutical Chemistry, Faculty of Pharmacy, Kafrelsheikh University, Kafrelsheikh, Egypt

**Keywords:** COVID-19, polymerase inhibitors, fragment-based drug design, molecular dynamics, MM-PBSA calculations

## Abstract

The world has recently been struck by the SARS-Cov-2 pandemic, a situation that people have never before experienced. Infections are increasing without reaching a peak. The WHO has reported more than 25 million infections and nearly 857,766 confirmed deaths. Safety measures are insufficient and there are still no approved drugs for the COVID-19 disease. Thus, it is an urgent necessity to develop a specific inhibitor for COVID-19. One of the most attractive targets in the virus life cycle is the polymerase enzyme responsible for the replication of the virus genome. Here, we describe our Structure-Based Drug Design (SBDD) protocol for designing of a new potential inhibitor for SARS-COV-2 RNA-dependent RNA Polymerase. Firstly, the crystal structure of the enzyme was retrieved from the protein data bank PDB ID (7bv2). Then, Fragment-Based Drug Design (FBDD) strategy was implemented using Discovery Studio 2016. The five best generated fragments were linked together using suitable carbon linkers to yield compound **MAW-22**. Thereafter, the strength of the binds between compound **MAW-22** and the SARS-COV-2 RNA-dependent RNA Polymerase was predicted by docking strategy using docking software. **MAW-22** achieved a high docking score, even more so than the score achieved by Remdesivir, indicating very strong binding between **MAW-22** and its target. Finally, three molecular dynamic simulation experiments were performed for 150 ns to validate our concept of design. The three experiments revealed that **MAW-22** has a great potentiality to inhibit the SARS-COV-2 RNA-dependent RNA Polymerase compared to Remdesivir. Also, it is thought that this study has proven SBDD to be the most suitable avenue for future drug development for the COVID-19 infection.

## Highlights

- Structure-Based drug design approach suggested **MAW-22** as a potential SARS-CoV-2 polymerase.
- **MAW-22** demonstrated strong binding affinity and energy profile for SARS-CoV-2 polymerase better than Remdesivir.
- **MAW-22** could be used as an effective agent for management of SARS-CoV-2 infection.
- Computer aided drug design is an efficient tool to develop drugs for SARS-CoV-2 infection.

## Introduction

The World Health Organization (WHO) declared COVID-19 as a pandemic disease on 11th March 2020. Since that time, COVID-19 has continued to expand and spread. The severe acute respiratory syndrome (SARS CoV-2) virus causing the COVID-19 infection has now affected 216 countries, with 25,803,688 confirmed cases and 857,766 deaths reported globally (as of 1st September 2020) (Carlos et al., 2020; Perlman, 2020; She et al., 2020). The prefix “Corona” comes from the Latin word for “crown,” this named for the virus' t crown-like appearance under electron microscope (Almeida et al., 1968; Tyrrell and Fielder, 2002). Coronaviruses, a family of RNA viruses in the order known as Nidovirales, are a positive-sense single-stranded RNA virus with medium size viruses ranging from 26 to 32 kb in length (Su et al., 2016). They are significant viral pathogens that affect animals and humans, causing viral pneumonia. There are four genera of coronaviruses (CoV): alpha, beta, gamma, and delta (Coronaviridae Study Group of the International Committee on Taxonomy of Viruses, 2020). Alpha and beta are responsible for seven coronaviruses that cause diseases in humans, while gamma and delta are pathogens in animals, not humans. SARS-COV-2 is the seventh coronavirus found to cause diseases in humans and is a novel Beta coronavirus (group 2B) (Zhu et al., 2020). Through genetic analysis, SARS-COV-2 has shown similarity to SARS-COV, with an 80% sequence homology similarity (Lu et al., 2020).

The first COVID-19 infections were estimated to be as early as November, 2019. Although studies are underway, it is still uncertain from where the SARS-CoV-2 pandemic began, however since December 2019, it has caused an outbreak of Acute Respiratory Distress syndrome (ARDS) and spread globally. On 30, January 2020, WHO declared a public health emergency due to SARS-COV-2. Studies showed that SARS-COV-2 originated from bats and translated to humans. Although the complete mechanism of transmission is unknown, it is likely that bats are the original source (Cavanagh, 2007). The subsequent cases involved family members of patients, healthcare workers, and finally human-to-human transmission (Chan et al., 2020). Moreover, transmission can be from symptomatic patients and also from asymptomatic individuals who are carrying the virus. Transmission could be conveyed through different modes of transmission. Although the exact modes are still unknown, it could be transmitted through respiratory droplets, and through exposure to sneezing or coughing from COVID-19 patients, through faces, and from close contact <2 m (Chan et al., 2020). COVID-19 disease and the common cold share common symptoms, including cough, fever, shortness of breath, and fatigue, which makes it confusing to differentiate between the two (Armstrong et al., 2019; Dong et al., 2020; Wrapp et al., 2020). SARS-CoV-2 attacks the lower respiratory system, like SARS-CoV and MERS, causing pneumonia, moreover it can also attack the central nervous system, kidney, liver, and gastrointestinal system, causing multiple organ damage (Zhu et al., 2020).

Two other coronaviruses from the last two decades, middle east respiratory syndrome (MERS) and severe acute respiratory syndrome (SARS), which happened in 2012 (Zaki et al., 2012) and 2003 (Chan-Yeung and Xu, 2003), respectively, had much lower health impacts compared to the contagious SARS-Cov-2 (Drosten et al., 2003; Ksiazek et al., 2003; Chan et al., 2020). This devastating virus could be the most threatening outbreak the world has faced as it could threaten the life we know. To date, numbers of cases are growing with no approved antiviral agent specific for coronaviruses in humans. Although attempts have been carried out using different approved antivirals and immunomodulatory agents, they have not demonstrated acceptable efficacy in randomized clinical trials (Wang et al., 2020).

The SARS-CoV-2 genome is about ~30,000 nucleotides. It encodes four structural proteins–Nucleocapsid (N) protein, Membrane (M) protein, Spike (S) protein, and Envelop (E) protein—and several non-structural proteins (nsp) that are essential in the virus replication cycle (21 new). The virus enters human cells by attaching its spike protein to the human angiotensin converting enzyme 2 (ACE2) receptors present at the surface of numerous cells, like those of the lungs and GIT. Then, the Spike protein is subjected to proteolytic cleavages by host proteases to release the fusion peptide. This is followed by a cascade of cellular processes that ends with virus entry into the cytoplasm. After that, the virus is uncoated, releasing its single-stranded RNA genome into cytoplasm where the replication and transcription take place by the aid of the virus' several non-structural proteins. Finally, the resulting proteins from the replication and transcription processes are assembled into new virions ready to infect new cells (Boopathi et al., 2020; Naqvi et al., 2020).

It is a difficult process to effectively develop specific COVID-19 direct acting antivirals (DAAs), however it is urgent to do so. From the above-mentioned life cycle of SARS-COV-2, several enzymes are considered potential targets for developing specific drugs against COVID-19, including non-structural protein 12 (nsp12), RNA Dependent RNA Polymerase (RdRp), 3C-like protease (3CL^pro^), Papin like protease (PL^Pro^), and human angiotensin converting enzyme 2 receptor (ACE2) (Wang et al., 2020). 3C-like protease (3CL^pro^) and Papin like protease (PL^Pro^) play crucial roles in the SARS-COV-2 replication cycle by processing the resulting polyprotein from the transcription stage into functioning subunits. Inhibiting any of the two proteases is believed to cease the virus replication cycle; blocking the angiotensin converting enzyme 2 receptor is also believed to prevent the virus entry by preventing the attachment of the SARS-COV-2 spike protein to the cell surface. Another attractive target to develop specific drugs for COVID-19 infection is the multi-subunit machinery SARS-COV-2 RNA-dependent RNA polymerase (Ziebuhr, 2005). The nsp12 RNA dependent RNA polymerase plays a central role in the transcription and replication process by catalyzing the synthesis of viral RNA of COVID-19 with the assistance of two essential cofactors: non-structural protein 7 (nsp7) and non-structural protein 8 (nsp8) (Subissi et al., 2014). This is the reason that the broad-spectrum antiviral Remdesivir, targeting nsp12, was given great attention as an RNA-dependent RNA polymerase inhibitor for COVID-19 infection, and studies show good results for treating COVID-19 viral disease with this. In early 2020, Remdesivir was tested as a potential treatment for COVID-19. It has been approved for use in many countries like the USA, Japan, the UK, and Singapore (Grein et al., 2020; Lamb, 2020; Mehta et al., 2020). It is highly recommended in severe symptoms and in emergency cases, as it would reduce the time needed for full recovery from the virus (Drożdżal et al., 2020; Mehta et al., 2020).

Most of the conducted studies to discover inhibitors for COVID-19 infection aimed either to repurpose already approved drugs or to design novel compounds for the above-mentioned potential targets, and many of those studies obtained good outcomes by implementing various Computer-aided drug discovery techniques like docking, molecular dynamics, and Fragment-based drug design (FBDD), proving the power and importance of these techniques in the process of drug discovery. In this work, we report on the employment of Computer-aided drug design studies into the identification of a novel inhibitor for RNA-dependent RNA polymerase inhibitor of SARS-COV-2 (Alamri et al., 2020; Choudhury, 2020; ul Qamar et al., 2020).

## Materials and Methods

### Fragment Based Drug Design (FBDD)

The crystal structure of SARS-COV-2 RNA-dependent RNA polymerase in complex with Remdesivir was retrieved from the protein data bank ID (7bv2) (Yin et al., 2020). A cavity surrounding the co-crystalized Remdesivir was constructed by Discovery studio 2016 (Dassault Systèmes BIOVIA, 2016). The cavity was extended to make good use of the entire active site. Then, a fragment-based approach was implemented using the *de novo* receptor strategy to fetch fragments from a library of fragments that fit properly into the active site of the enzyme (Böhm, 1992). The default Ludi fragments library found in the discovery studio, which contains 1,053 diverse fragments with molecular weights less than (300 KD), was used as the input source of fragments. The strength of binding of the retrieved fragments from the search was evaluated by docking to the receptor cavity using MCSS (Multiple Copy Simultaneous Search) (Caflisch et al., 1993; Evensen et al., 1997). Finally, the successful fragments were linked together with suitable carbon linkers to produce compound **MAW-22 (**[(2S)-3-[2-amino-5-[(1R)-1-[6-amino-4-(dihydroxymethylene)-1H-pyridin-2-yl]propyl]-4-pyridyl]-2-carboxy-2-[(2-carboxybenzoyl)amino]propyl]-[(1S)-1-carboxypropyl]ammonium**)**. The binding strength of the generated compound was evaluated from the docking stage.

### *In silico* ADME and toxicity Calculations

The online Swiss ADME server (http://www.swissadme.ch/index.php) and Preadmet server (https://preadmet.bmdrc.kr/) were implemented to calculate the compound's physicochemical properties and toxicity, respectively.

### Docking

The SARS-COV-2 RNA-dependent RNA polymerase retrieved from the protein data bank was utilized to conduct the docking study using Discovery Studio 2016. The active site was determined from the binding of Remdesivir by constructing a cavity surrounding the binding domain of the co-crystalized Remdesivir. The receptor was prepared by protein preparation wizard, while the ligands were prepared by the ligand preparation wizard. The receptor was energy minimized and equilibrated for 10 Nano seconds (ns) under GROMOS96 43a1 force field (see molecular dynamic section). Docking was commenced by C-Docker software found in the discovery Studio 2016 package in two steps (Wu et al., 2003). Step one involved the re-docking of Remdesivir to its corresponding receptor and the second step was the docking of the generated compound **MAW-22** from a fragment-based drug design stage. The docking results were visualized and analyzed by the Discovery studio visualizer available from Biovia Inc^1^.

### Molecular Dynamics

In the current study, we performed three molecular dynamic simulation experiments to support our concept of design. One experiment was conducted using the polymerase alone (none complexed with any ligand). The second and third experiments were for SARS-COV-2 RNA-dependent RNA polymerase enzyme in complex with Remdesivir and with the generated compound **MAW-22**, respectively. The entire MD simulation experiments were conducted using the latest version of GROningen MAchine for Chemical Simulations (GROMACS 2020.3) (Abraham et al., 2015). The receptor topology was obtained by the “pdb2gmx” script, while the ligand topologies were obtained by the CHARMM General Force Field CGENFF server and converted to the gromacs format using the “cgenff_charmm2gmx_py3_nx2.py” script (Phillips et al., 2005). Each of the generated ligand topologies was rejoined to the processed receptor structure to construct the ligand-protein complex. GROMOS96 43a1 force field was used to obtain the energy minimized conformations of all the processed complexes (Chiu et al., 2009). After that, those complexes were solvated with a single point charge (SPC) water model to add water molecules to the cubic simulation boxes. Neutralization of the system net charges was done by adding counter-ions using the “gmx genion” script. Energy minimization of the unbound enzyme and the two complexes was achieved by employing the steepest descent minimization algorithm with a maximum of 50,000 steps and <10.0 kJ/mol force. Then, the solvated energy minimized structures were equilibrated with two consecutive steps. Firstly, NVT ensemble with a constant number of particles, volume, and temperature was done for 2 ns, followed by NPT ensemble with a constant number of particles, pressure, and temperature for 8 ns. In the two systems, only the solvent molecules were allowed free movement to ensure its equilibration in the system, while other atoms were restrained. A constant temperature of 310 K and constant pressure of 1 atm were maintained through the entire MD simulation. The long-range electrostatic interactions were obtained by the particle mesh Eshwald method with a 12 Å cut-off and 12 Å Fourier spacing (Bhardwaj et al., 2020). Finally, the three well-equilibrated systems (one empty protein and two protein-ligand complexes) entered the production stage without any restraints for 150 ns with a time step of 2 fs, and after every 5 ps the structural coordinates were saved. The root mean square deviation (RMSD) was calculated from the generated trajectories of the MD simulations as well as the distances of the formed hydrogen bonds between the receptor and the ligands by various scripts of GROMACS.

### MM-PBSA calculation

A common application in MD simulations and thermodynamic calculations is to determine the binding free energy of a protein-ligand complex. Generally, the binding free energy of protein and ligand complexes can be calculated using the molecular Mechanic/Poisson-Boltzmann Surface Area (MM-PBSA) alongside MD simulations using the following equation:

$$\begin{array}{l} \Delta {\text{G}}_{\text{(Binding})}{\text{= G}}_{\text{(Complex})}{-\text{ G}}_{\text{(Receptor})}{-\text{ G}}_{\text{(5Ligand})} \end{array}$$

Where G (complex) is the total free energy of the protein–ligand complex and G (receptor) and G (ligand) are total free energies of the isolated protein and ligand in solvent, respectively. The total free energy of any of the three mentioned entities (complex or receptor or ligand) could be calculated from its molecular mechanics potential energy plus the energy of solvation. Thus, the “g_mmpbsa” (Kumari and Kumar, 2014) package of GROMACS was used to perform MM-PBSA calculations through all the MD trajectories.

## Results and Discussion

### FBDD

Fragment-based drug discovery is a very important technique in the field of drug design (de Kloe et al., 2009). The technique enables the discovery of novel drugs through the screening of fragments databases. The fragments should always be low in molecular weight, less than (300 KD), and chemically diverse (Kumar et al., 2012). FBDD is advantageous over other high throughput screening (HTS) methods because of three points. The first advantage is that the active site is better covered and fully explored in FBDD. This is attributed to small fragments that could be easily inserted in any space in the active site in contrast to large molecules from other HTS methods (Blum and Reymond, 2009; Roughley and Hubbard, 2011). The second point is that screening a fragment library achieves higher hit rates as compared to conventional HTS (Mortenson and Murray, 2011). However, in this study our focus was to design one compound with the maximum binding strength. The last advantage of FBDD over HTS is that compounds designed by FBDD achieve higher binding affinity than compounds designed by HTS (Kumar et al., 2012).

It is well-established that there are three strategies in FBDD: (a) Fragment growing (Kirsch et al., 2019), in which a fragment that obeys the role of three is increased in size to optimize the interaction of the proposed target; (b) Fragment merging (Kirsch et al., 2019), in which two fragments bound to the same regions in the binding site of the target are merged to give one compound; and (c) Fragment linking (Kirsch et al., 2019), in which two or more fragments bound to different regions in the binding site of the target are linked together by a suitable linker to yield one compound. The active site was determined by a cavity surrounding the binding of Remdesivir. The *de novo* receptor wizard available in the discovery studio 2016 was employed to screen the fragment library. The technique subdivides the cavity, and all the fragments are screened through the entire binding domain (cavity). Energy estimate 3 was used as a scoring function. Successful fragments should achieve good interaction with the receptor and also make a negative change in the free energy upon binding to the receptor. The conducted search resulted in 821 fragments. Those fragments were further filtered through docking to the receptor cavity using the MCSS protocol. The protocol involves the following steps (Evensen et al., 1997): generating conformations for each fragment, screening of all the fragments through the binding cavity, CHARMM fragment minimization, and clustering and removing fragments that converge to similar positions. The resulted top fragment in each region in the binding site was selected to construct **MAW-22**.

**Figure 1:**
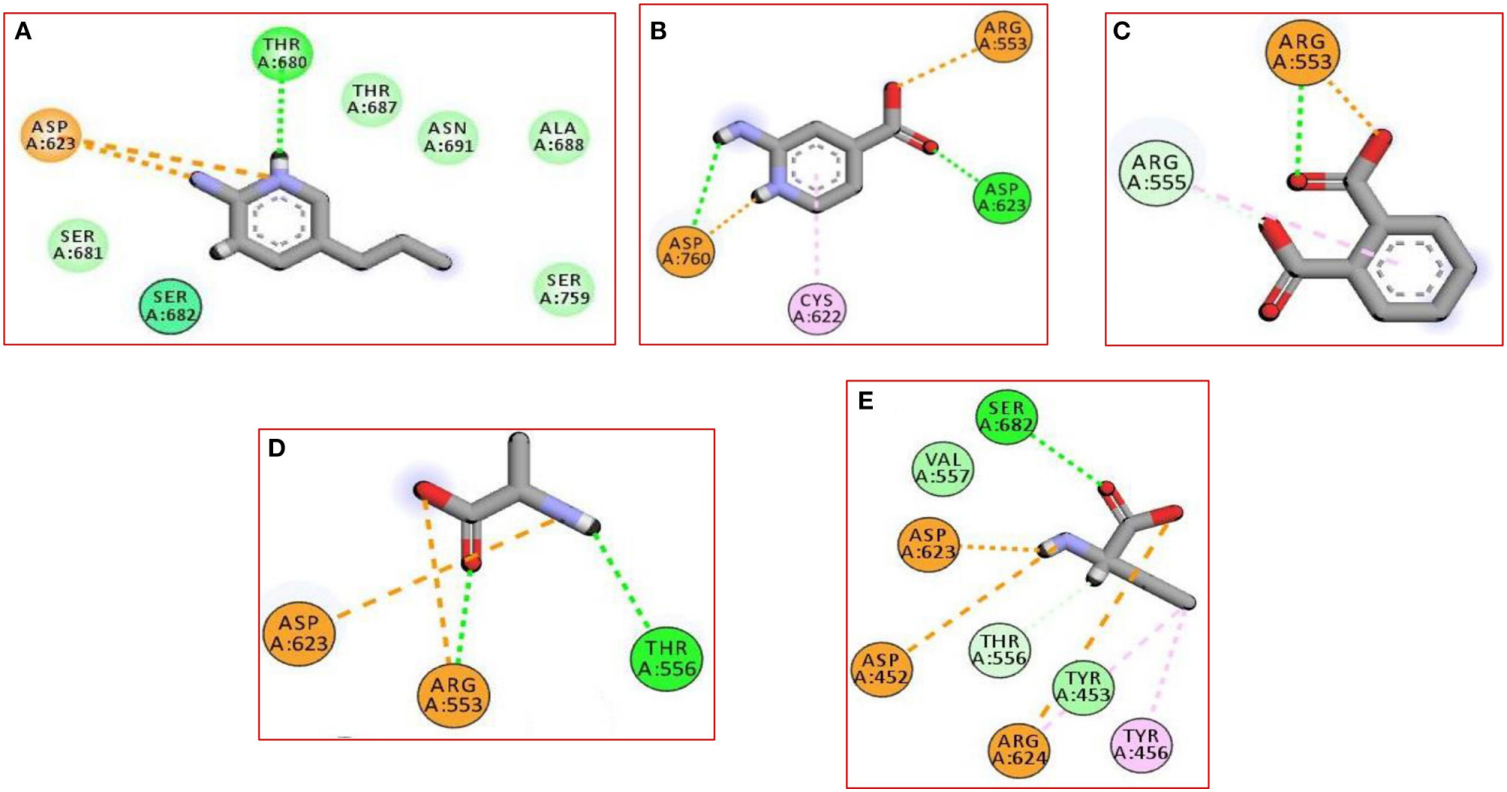
The 2D interaction diagram for the five produced fragments from FBDD **(A)** fragment 1 **(B)** fragment 2 **(C)** fragment 3 **(D)** fragment 4 **(E)** fragment 5.

**Figure 2:**
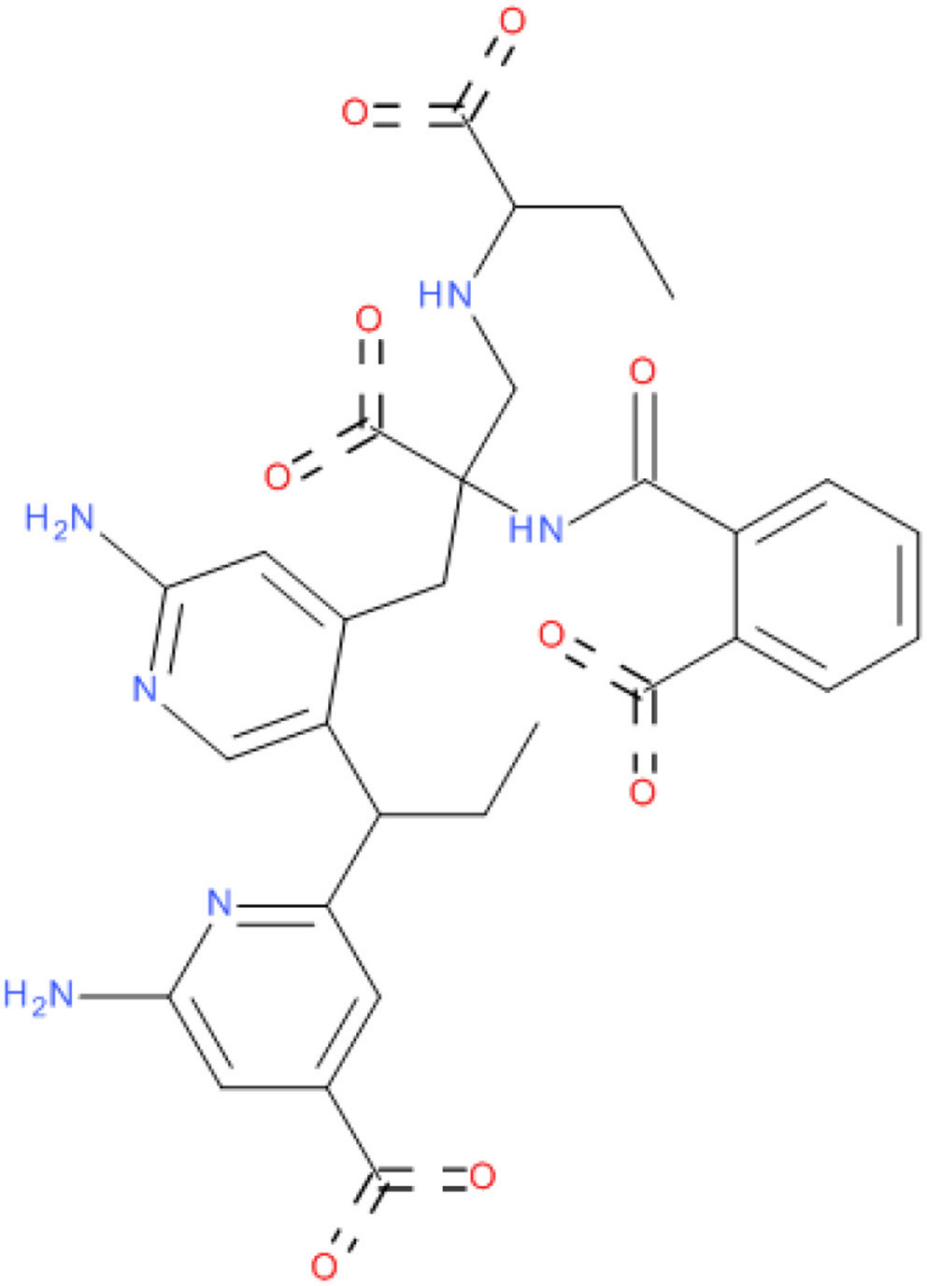
2D structure of compound **MAW-22**.

In the current work, we did not have a lead compound or a fragment to apply the Fragment-growing approach, whereas the generated compound was designed from scratch. Also, all generated fragments were apart from each other, so the fragment linking strategy was more suitable than the fragment merging strategy. The binding of the selected fragments was strong and involved many types of interactions (Figure 1). Those fragments were then linked *via* a carbon linker to yield **MAW-22** (Figure 2).

### *In silico* ADME and toxicity Calculations

Any compound to be considered as a potential drug candidate should have acceptable pharmacokinetic and pharmacodynamic profiles, as well as a high safety margin. Thus, both Swiss ADME and Preadmet servers were employed to predict the physicochemical properties and potential toxicity of **MAW-22**. In general, both servers predicted excellent safety profiles of compound **MAW-22**, with no mutagenicity or carcinogenicity as predicted by the Preadmet server. On the other hand, Swiss ADME revealed that **MAW-22** has no inhibitory effect toward any hepatic Cytochrome enzyme, and thus it has neither hepatotoxicity nor drug-drug interactions and could be used concurrently with any drugs in a COVID-19 treatment protocol. Furthermore, both servers predicted no BBB penetration for **MAW-22**, so it could be safely used with no concerns about potential neurotoxicity. Another important advantage is that **MAW-22** would mostly have no teratogenic effect. This assumption is evidenced by that fact that placental penetration requires compounds to have molecular weights <500 Dalton, high Lipophilicity, and predominance of the non-ionized form (Griffiths and Campbell, 2015). MAW-22 is a polar compound with logP equals−0.13 and molecular weight of 630 Dalton, as well as four ionized carboxylate groups. Thus, it would probably have no penetration with significant concentration through the placenta and could be safely given to pregnant woman with a COVID-19 infection. **MAW-22** violated the Lipinski rule only in the Molecular weight and the number of hydrogen bond donors. But when it comes to medicinal chemistry friendliness, **MAW-22** has no alertness for either PAINS or Brenk (Veber et al., 2002; Baell and Holloway, 2010).

### Docking

**Figure 3:**
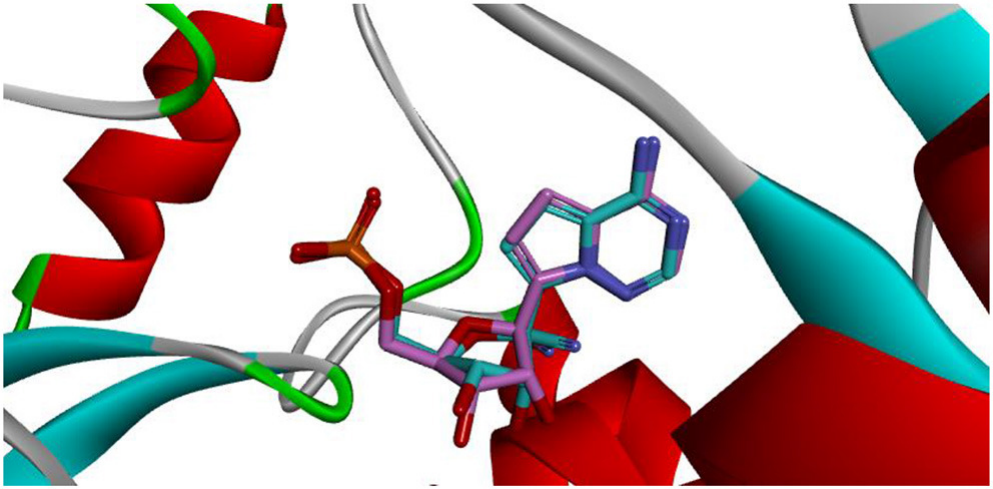
Superimposition between co-crystalized (pink) and re-docked pose (cyan) of Remdesivir showing nearly the same binding mode.

Docking is the most widely used technique in drug design. In the current study, docking was inevitable as it is the only computational method able to predict the exact binding between the generated compound **MAW-22** and the SARS-COV-2 RNA-dependent RNA Polymerase. Yet there are two issues; the first is that docking is vulnerable to error and needs proper validation, and the second is that the docking results need to be compared to an experimental reference. These two points were addressed by re-docking Remdesivir to the SARS-COV-2 RNA-dependent RNA Polymerase. The calculated RMSD between the docked and co-crystalized poses of Remdesivir was 0.48, indicating a valid docking approach (Figure 3).

**Figure 4:**
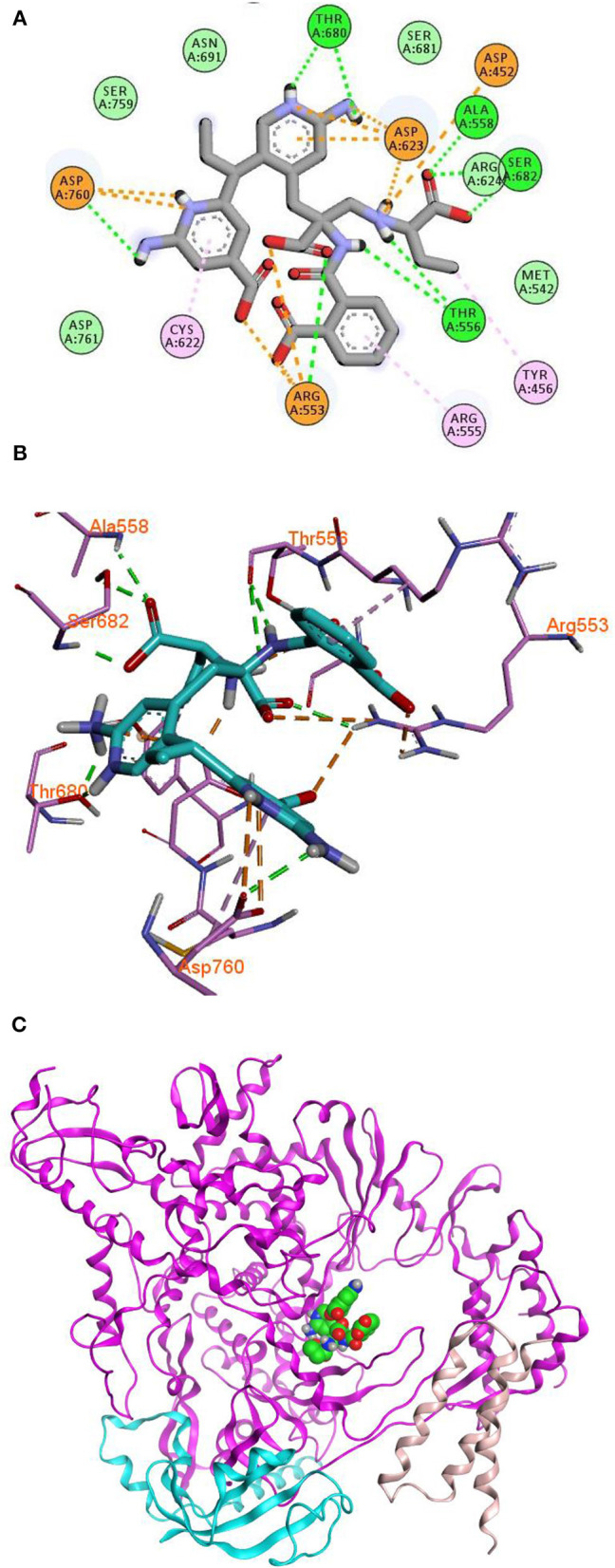
2D **(A)**, 3D **(B)** interaction diagram **(C)** binding of **MAW-22** to the active site of SARS-COV-2 RNA-Dependent RNA Polymerase.

Remdesivir achieved a—C-Docker _Energy score of 87.3, whereas **MAW-22** achieved a—C-Docker _Energy score of 135.7. The high score achieved by **MAW-22** is well-matched with its strong binding and interactions with the SARS-COV-2 RNA-dependent RNA Polymerase. As shown in Figure 4, **MAW-22** was able to engage in a number of diverse interaction types with its target. For instance, the proposed compound was involved in hydrogen-bond interactions with residues THR556, ALA558, ARG553, THR680, SER682, and ASP760, in addition to ionic interactions with residues ASP452, ASP623, ASP760, and ARG553. Moreover, **MAW-22** achieved several hydrophobic interactions with residues CYS622, ARG555, and TYR456. The detailed interactions of **MAW-22** within the SARS-COV-2 RNA-dependent RNA Polymerase active site were summarized in Table 1.

**Table 1:** Types of interactions of **MAW-22** within COVID-19 polymerase active site, and distance (A°).

**Bond type**	**Distance A^**°**^**	**Bond type**	**Distance A^**°**^**
Hydrogen bond with Alanine 558	2.13	Ionic interaction with Aspartic acid 760	3.20
Hydrogen bond with Serine 682	2.04	Ionic interaction with Aspartic acid 623	1.78
Hydrogen bond with Serine 682	1.94	Ionic interaction with Aspartic acid 623	3.90
Hydrogen bond with Threonine 556	2.27	Ionic interaction with Aspartic acid 623	4.08
Hydrogen bond with Threonine 556	2.96	Ionic interaction with Aspartic acid 623	4.36
Hydrogen bond with Arginine 553	2.36	Ionic interaction with Arginine 553	2.12
Hydrogen bond with Aspartic acid 760	2.30	Ionic interaction with Arginine 553	4.70
Hydrogen bond with Threonine 680	1.68	Ionic interaction with Arginine 553	2.97
Hydrogen bond with Threonine 680	1.86	Pi-Alkyl interaction with Arginine 555	4.92
Ionic interaction with Aspartic acid 452	4.92	Pi-Alkyl interaction with Cysteine 622	5.04
Ionic interaction with Aspartic acid 760	3.07	Pi-Alkyl interaction with Tyrosine 456	4.57

### Molecular Dynamics

Molecular dynamic simulations, such as identification of potential inhibitors for promising targets, studying the nature of macromolecules, or interpretations of drug resistances, have been implemented in many drug discovery applications (El-Hasab et al., 2018; El-Hassab et al., 2019; Alamri et al., 2020; Nagarajan et al., 2020). Despite the good outcomes from the docking study that supported our rationale of design, molecular dynamic simulation experiments were conducted for extra confirmation and validation for the entire work. Also aiming to identify and study the nature of the SARS-COV-2 RNA-dependent RNA Polymerase to give insights for future lead optimization, we performed three dynamic simulations, one for the free SARS-COV-2 RNA-dependent RNA Polymerase and the two others for the enzyme with Remdesivir and compound **MAW-22**.

### RMSD and RMSF Analysis and Hydrogen Bond Monitoring

**Figure 5:**
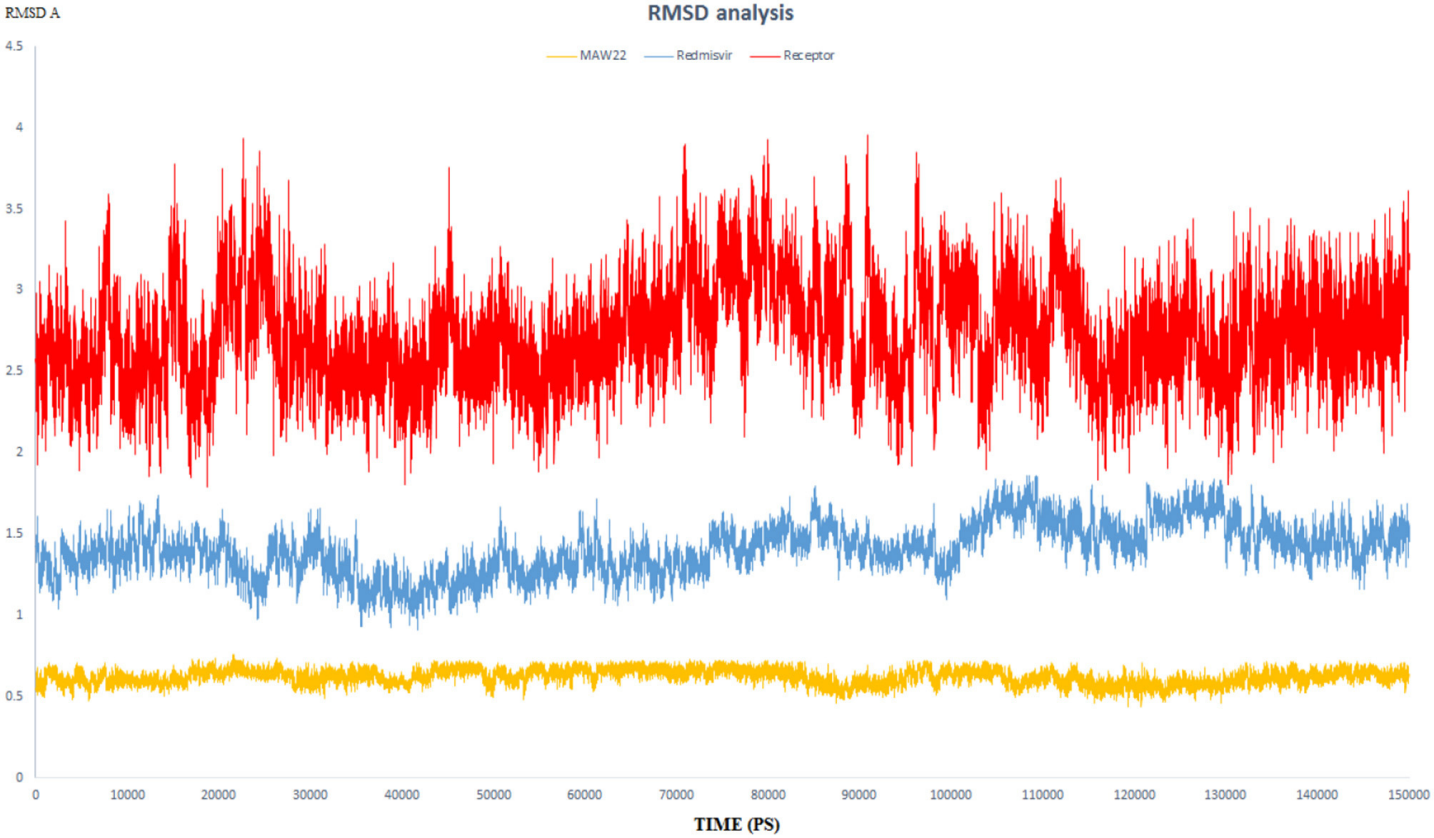
The RMSD of three dynamic simulation experiments. Red color represents SARS-COV-2 RNA-dependent RNA Polymerase without a ligand; blue line represents SARS-COV-2 RNA-dependent RNA Polymerase complex with Remdesivir, and orange line represents SARS-COV-2 RNA-dependent RNA Polymerase complex with **MAW-22**.

**Figure 6:**
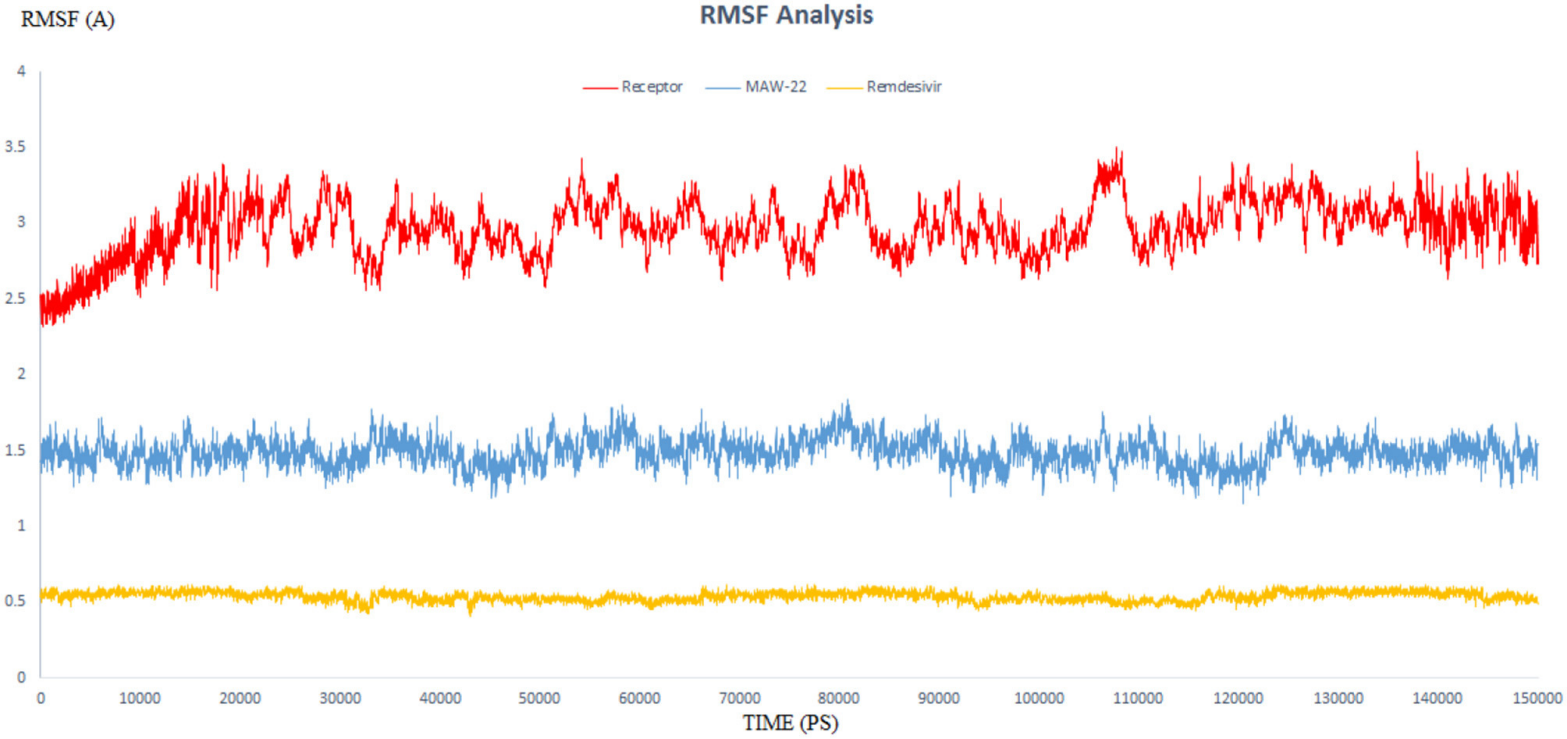
The RMSF of three dynamic simulation experiments. Red color represents SARS-COV-2 RNA-dependent RNA Polymerase without a ligand; blue line represents SARS-COV-2 RNA-dependent RNA Polymerase complex with Remdesivir, and orange line represents SARS-COV-2 RNA-dependent RNA Polymerase complex with **MAW-22**.

The endeavor of any viral polymerase enzyme is to replicate the virus genome or polyproteins, a function that needs a wide range of flexibility at least in the active site to accommodate both the template and the replicate (Kennedy et al., 2007). All the reported polymerases are relatively dynamic and have a wide active site (Bose-Basu et al., 2004). So, the conducted simulation experiment aimed to determine the extent of dynamicity of the SARS-COV-2 RNA-dependent RNA Polymerase enzyme as well as to be a reference for comparison with the other two simulation experiments. The calculated RMSD and RMSF of all the residues of the unbound enzyme reached 3.96 and 3.50 A°, respectively, revealing the high dynamic properties of the SARS-COV-2 RNA-dependent RNA Polymerase (Figures 5, 6).

This dynamicity and instability of the enzyme fit perfectly for its intended role to duplicate the entire RNA genome of the virus. This means that the active site of the SARS-COV-2 RNA-dependent RNA Polymerase needs to accommodate not only for the template RNA but also for the generated duplicate before releasing them and starting another replication process. Also, this dynamicity gives us a reliable parameter to evaluate the efficiency of a proposed inhibitor, as potential potent inhibitors should have the ability to bind strongly to the enzyme and form stable non-dynamic complex. Thus, it was important to monitor the dynamic behavior for both SARS-COV-2 RNA-dependent RNA Polymerase complex with Remdesivir and SARS-COV-2 RNA-dependent RNA Polymerase complex with **MAW-22** through measuring the RMSD and RMSF for both the complexes. This is far more reliable than the static binding image or the energy score provided from the docking. RMSD and RMSF from MD simulations are more reliable indicators to monitor the stability of the protein-ligand complex and could verify the predicted binding mode.

RMSD values for both **MAW-22** and Remdesivir reached their maximum dynamicity peaks at 0.76 and 1.86 A°, respectively (Figure 5), whereas the RMSF values were 0.62 A° for **MAW-22** and 1.84 A° for Remdesivir at their maximum fluctuation (Figure 6). This indicates that **MAW-22** has a higher ability to inhibit SARS-COV-2 RNA-dependent RNA Polymerase than Remdesivir. The potential inhibitory activity of **MAW-22** may be attributed to its strong binding ability and many involved interactions formed between **MAW-22** and its target COVID-19 polymerase. Thus, it was worthy to monitor the stability of those interactions through an MD experiment. GROMACS has built-in commands that were used to measure the distances of the formed hydrogen bonds between **MAW-22** and COVID-19 polymerase. The distance between the hydrogen bond donor and the hydrogen bond acceptor in a valid hydrogen bond should always be <3.5 A°. This criterion was fulfilled in all the formed hydrogen bonds between **MAW-22** and COVID-19 polymerase, indicating a stable and valid binding between **MAW-22** and its target (Table 2).

**Table 2:** The average distances of all the hydrogen bond formed between the **MAW-22** and Covid-19 polymerase through the entire 150 ns MD simulation.

**Hydrogen bond name**	**Average distance (A^**0**^) ± SD**
Hydrogen bond with Alanine 558	2.15 ± 0.11
Hydrogen bond with Serine 682	2.0 ± 0.07
Hydrogen bond with Serine 682	2.05 ± 0.15
Hydrogen bond with Threonine 556	2.21 ± 0.1
Hydrogen bond with Threonine 556	3.0 ± 0.09
Hydrogen bond with Arginine 553	2.32 ± 0.12
Hydrogen bond with Aspartic acid 760	2.37 ± 0.1
Hydrogen bond with Threonine 680	1.85 ± 0.2
Hydrogen bond with Threonine 680	1.8 ± 0.13

### MM-PBSA Binding Free Energy Calculations

The g_mmpbsa package was used to calculate the MM-PBSA binding free energy for the two complexes—**MAW-22** and Remdesivir—bound to SARS-COV-2 RNA-dependent RNA Polymerase enzyme by the employment of MmPbSaStat.py python script. n script allows the package to calculate the total free energy for each component of the complex, i.e., the energy of the complex, receptor, and the ligand. Furthermore, the free energy for each component could be calculated using the cumulative sum of its molecular mechanics' potential energy in a vacuum and the free energy of solvation. Molecular mechanics' potential energy includes the energy of both bonded as well as non-bonded interactions (Vanderwal's and electrostatic interaction energies), while the free energy of solvation includes the polar solvation energy (electrostatic) and non-polar solvation energy (non-electrostatic) (Kumari and Kumar, 2014). One of the most widely used non-polar models is the solvent accessible surface area (SASA) (Kumari and Kumar, 2014). All those types of energies were calculated by the g_mmpbsa package, along with the values standard deviation, and then summed together to yield the average total free energy of each component. Finally, the binding free energy could be calculated by subtracting the total free energy of the receptor and the total free energy of the ligand from the total free energy of the complex. As a general fact, the lesser the binding free energy the more stable the complex and the stronger the binding between the ligand and the receptor. Table 3 summarizes the interaction energies and the binding free energy for both the complexes.

**Table 3:** MM-PBSA calculations of the binding free energy for the two complexes; **MAW-22** and Remdesivir.

**Complex**	**ΔE _**binding (kj/mol)**_**	**ΔE _**Bonded interaction (kj/mol)**_**	**ΔE _**Electrostatic (kj/mol)**_**	**ΔE _**VanderWaal(*kj*/*mol*)**_**	**ΔE _**polar solvation (kj/mol)**_**	**SASA _**(kJ/mol)**_**
**MAW-22**	−390.24 ± 20.501	1.35 ± 0.125	−134.703 ± 20.105	−343.405 ± 25.42	117.722 ± 16.104	−31.204 ± 1.092
Remdesivir	−328.447 ± 22.334	3.547 ± 0.232	−118.958 ± 22.612	−294.110 ± 26.713	106.352 ± 18.001	−25.278 ± 2.08

Generally, **MAW-22**–Covid-19 complex was better than Remdesivir complex in all the calculated energy formats, except for polar solvation energy; its average binding free energy reached −390.24 Kj/mol, while Remdesivir's average binding free energy reached −328.447 Kj/mol. The overall results of the three dynamic simulations supported our concept of design and validated the entire virtual screening approach; also, they emphasized and assured the potential inhibitory effect of **MAW-22** on COVID-19 polymerase enzyme.

## Future Outlook

The aim of our study was not only to design a potential inhibitor for the devastating COVID-19 infection but also to establish guidance that could open a new era for the development of an effective treatment for COVID-19. Despite our success in designing a potential inhibitor for SARS-COV-2 RNA-dependent RNA Polymerase, a lot of work is still needed and further optimization for the proposed hit compound should be done before reaching the clinic. Regardless, this study established the key residues and the required type of bonds for inhibiting the SARS-COV-2 RNA-dependent RNA Polymerase enzyme. For instance, compound **MAW-22** has a good combination of basic and acidic groups complementary to the active site of the enzyme that has been found to be rich in basic amino acids like Arginine and Lysine and acidic amino acids like Aspartic acid. Thus, compounds that have negatively and/or positively ionizable groups would have a great chance to bind strongly and inhibit the SARS-COV-2 RNA-dependent RNA Polymerase enzyme. So, drug design techniques that identify the most suitable function groups, such as 3D Pharmacophore, should be applied for further improvement of the generated hit compound. Also, further optimization of the ADMET profile and drug likeness should be considered. Based on the outcomes from this study, our future work will involve the implementation of more structure-based drug design strategies to furnish a lead compound that is worthy of entering clinical trials for the treatment of COVID-19.

## Conclusion

In this study we employed a protocol of structure-based drug design with the primary aim of designing a potential specific inhibitor for SARS-COV-2 RNA-dependent RNA Polymerase enzyme. The crystal structure of SARS-COV-2 RNA-dependent RNA Polymerase was retrieved from the protein data bank PDB ID (7bv2) in complex with Remdesivir. Firstly, FBDD strategy was implemented using *de novo* Receptor wizard found in the Discovery Studio 2016. The default fragment database of the software was used to identify potential fragments that could interact strongly with the active site of SARS-COV-2 RNA-dependent RNA Polymerase enzyme. The five best generated fragments were linked together using suitable carbon linkers to yield compound **MAW-22**. Thereafter, the strength of binding between compound **MAW-22** and the SARS-COV-2 RNA-dependent RNA Polymerase enzyme was predicted by Docking strategy using C-Docker software. Compound **MAW-22** achieved a high score of docking, even more so than the score achieved by Remdesivir, which indicates a very strong binding between **MAW-22** and the SARS-COV-2 RNA-dependent RNA Polymerase enzyme. Finally, three molecular dynamic simulation experiments were performed for 150 ns to validate and augment our concept of design. The three experiments revealed that compound **MAW-22** has a great potentiality to inhibit the SARS-COV-2 RNA-dependent RNA Polymerase enzyme, even more so than Remdesivir. The aim of this study was not only to design a potential inhibitor but also to establish guidance for future drug development for the COVID-19 infection.

## Data Availability Statement

Publicly available datasets were analyzed in this study. This data can be found here: Protein Data Bank ID (7bv2).

## Author Contributions

All authors listed have made a substantial, direct and intellectual contribution to the work, and approved it for publication.

## Conflict of Interest

The authors declare that the research was conducted in the absence of any commercial or financial relationships that could be construed as a potential conflict of interest.

## Footnotes

^1^https://3dsbiovia.com/resource-center/downloads/

## References

[B1] Abraham M. J. Murtola T. Schulz R. Pall S. Smith J. C. Hess B. (2015). GROMACS: High performance molecular simulations through multi-level parallelism from laptops to supercomputers. SoftwareX 1–2, 19–25. 10.1016/j.softx.2015.06.001

[B2] Alamri M. A. Tahir ul Qamar M. Mirza M. U. Bhadane R. Alqahtani S. M. Muneer I. (2020). Pharmacoinformatics and molecular dynamics simulation studies reveal potential covalent and FDA-approved inhibitors of SARS-CoV-2 main protease 3CLpro. J. Biomol. Struct. Dyn. 23, 1–3. 10.1080/07391102.2020.1782768 PMC7332866 32579061

[B3] Almeida J. D. Berry D. M. Cunningham C. H. Hamre D. Hofstad M. S. Mallucci L. (1968). Virology: coronaviruses. Nature 220:650 10.1038/220650b0

[B4] Armstrong G. L. MacCannell D. R. Taylor J. Carleton H. A. Neuhaus E. B. Bradbury R. S.. (2019). Pathogen genomics in public health. N. Engl. J. Med. 381, 2569–2580. 10.1056/NEJMsr1813907 31881145 PMC7008580

[B5] Baell J. B. Holloway G. A. (2010). New substructure filters for removal of pan assay interference compounds (PAINS) from screening libraries and for their exclusion in bioassays. J. Med. Chem. 53, 2719–40. 10.1021/jm901137j 20131845

[B6] Bhardwaj V. K. Singh R. Sharma J. Rajendran V. Purohit R. Kumar S. (2020). Identification of bioactive molecules from Tea plant as SARS-CoV-2 main protease inhibitors. J. Biomol. Struct. Dyn. 8, p1–13. 10.1080/07391102.2020.1766572 32397940 PMC7256349

[B7] Blum L. C. Reymond J. L. (2009). 970 million druglike small molecules for virtual screening in the chemical universe database GDB-13. J. Am. Chem. Soc. 131, 8732–8733. 10.1021/ja902302h 19505099

[B8] Böhm H. J. (1992). The computer program LUDI: a new method for the de novo design of enzyme inhibitors. J. Comput. Aided Mol. Des. 6, 61–78. 10.1007/BF00124387 1583540

[B9] Boopathi S. Poma A. B. Kolandaivel P. (2020). Novel 2019 coronavirus structure, mechanism of action, antiviral drug promises and rule out against its treatment. J. Biomol. Struct. Dyn. 29, 1–10. 10.1080/07391102.2020.1758788 32306836 PMC7196923

[B10] Bose-Basu B. DeRose E. F. Kirby T. W. Mueller G. A. Beard W. A. Wilson S. H.. (2004). Dynamic characterization of a DNA repair enzyme: NMR studies of [methyl-13C] methionine-labeled DNA polymerase β. Biochemistry 43, 8911–8922. 10.1021/bi049641n 15248749

[B11] Caflisch A. Miranker A. Karplus M. (1993). Multiple copy simultaneous search and construction of ligands in binding sites: application to inhibitors of HIV-1 aspartic proteinase. J. Med. Chem. 36, 2142–2167. 10.1021/jm00067a013 8340918

[B12] Carlos W. G. Dela Cruz C. S. Cao B. Pasnick S. Jamil S. (2020). COVID-19 disease due to SARS-CoV-2 (novel coronavirus). Am. J. Respir. Crit. Care Med. 201, P7–8. 10.1164/rccm.2014P7 32004066

[B13] Cavanagh D. (2007). Coronavirus avian infectious bronchitis virus. Vet. Res. 38, 281–297. 10.1051/vetres:2006055 17296157

[B14] Chan J. F. Yuan S. Kok K. H. To K. K. Chu H. Yang J.. (2020). A familial cluster of pneumonia associated with the 2019 novel coronavirus indicating person-to-person transmission: a study of a family cluster. Lancet 395, 514–523. 10.1016/S0140-6736(20)30154-9 31986261 PMC7159286

[B15] Chan-Yeung M. Xu R. H. (2003). SARS: epidemiology. Respirology 8, S9–14. 10.1046/j.1440-1843.2003.00518.x 15018127 PMC7169193

[B16] Chiu S. W. Pandit S. A. Scott H. L. Jakobsson E. (2009). An improved united atom force field for simulation of mixed lipid bilayers. J. Phys. Chem. B 113, 2748–2763. 10.1021/jp807056c 19708111

[B17] Choudhury C. (2020). Fragment tailoring strategy to design novel chemical entities as potential binders of novel corona virus main protease. J. Biomol. Struct. Dyn. 18, 1–5. 10.1080/07391102.2020.1771424 32452282 PMC7284137

[B18] Coronaviridae Study Group of the International Committee on Taxonomy of Viruses (2020). The species Severe acute respiratory syndrome-related coronavirus: classifying 2019-nCoV and naming it SARS-CoV-2. Nat. Microbiol. 5, 536–544. 10.1038/s41564-020-0695-z 32123347 PMC7095448

[B19] Dassault Systèmes BIOVIA BIOVIA Workbook Release. (2016). BIOVIA Pipeline Pilot. San Diego, CA: Dassault Systèmes.

[B20] de Kloe G. E. Bailey D. Leurs R. de Esch I. J. (2009). Transforming fragments into candidates: small becomes big in medicinal chemistry. Drug Discov. Today 14, 630–646. 10.1016/j.drudis.2009.03.009 19443265

[B21] Dong Y. Mo X. Hu Y. Qi X. Jiang F. Jiang Z. (2020). Epidemiological characteristics of 2143 pediatric patients with 2019 coronavirus disease in China. Pediatrics 145:e20200702 10.1542/peds.2020-0702 32179660

[B22] Drosten C. Günther S. Preiser W. Van Der Werf S. Brodt H. R. Becker S.. (2003). Identification of a novel coronavirus in patients with severe acute respiratory syndrome. N. Engl. J. Med. 348, 1967–1976. 10.1056/NEJMoa030747 12690091

[B23] Drożdżal S. Rosik J. Lechowicz K. Machaj F. Kotfis K. Ghavami S.. (2020). FDA approved drugs with pharmacotherapeutic potential for SARS-CoV-2 (COVID-19) therapy. Drug Resist. Updat. 53:100719. 32717568 10.1016/j.drup.2020.100719 PMC7362818

[B24] El-Hasab M. A. El-Bastawissy E. E. El-Moselhy T. F. (2018). Identification of potential inhibitors for HCV NS3 genotype 4a by combining protein–ligand interaction fingerprint, 3D pharmacophore, docking, and dynamic simulation. J. Biomol. Struct. Dyn. 36, 1713–1727. 10.1080/07391102.2017.1332689 28531373

[B25] El-Hassab M. A. El-Bastawissy E. E. El-Moselhy T. F. (2019). Identification of potential inhibitors for HCV NS5b of genotype 4a by combining dynamic simulation, protein–ligand interaction fingerprint, 3D pharmacophore, docking and 3D QSAR. J. Biomol. Struct. Dyn. 38, 4521–4535. 10.1080/07391102.2019.1685005 31647392

[B26] Evensen E. Joseph-McCarthy D. Karplus M. (1997). MCSS Version 2.1. Cambridge, MA: Harvard University.

[B27] Grein J. Ohmagari N. Shin D. Diaz G. Asperges E. Castagna A.. (2020). Compassionate use of remdesivir for patients with severe Covid-19. N. Engl. J. Med. 382, 2327–2336. 32275812 10.1056/NEJMoa2007016 PMC7169476

[B28] Griffiths S. K. Campbell J. P. (2015). Placental structure, function and drug transfer. Continu. Educ. Anaesth. Crit. Care Pain 15, 84–89. 10.1093/bjaceaccp/mku013

[B29] Kennedy W. P. Momand J. R. Yin Y. W. (2007). Mechanism for *de novo* RNA synthesis and initiating nucleotide specificity by t7 RNA polymerase. J. Mol. Biol. 370, 256–268. 10.1016/j.jmb.2007.03.041 17512007

[B30] Kirsch P. Hartman A. M. Hirsch A. K. Empting M. (2019). Concepts and core principles of fragment-based drug design. Molecules 24:4309. 10.3390/molecules24234309 31779114 PMC6930586

[B31] Ksiazek T. G. Erdman D. Goldsmith C. S. Zaki S. R. Peret T. Emery S.. (2003). A novel coronavirus associated with severe acute respiratory syndrome. N. Engl. J. Med. 348, 1953–1966. 10.1056/NEJMoa030781 12690092

[B32] Kumar A. Voet A. Zhang K. Y. (2012). Fragment based drug design: from experimental to computational approaches. Curr. Med. Chem. 19, 5128–5147. 10.2174/092986712803530467 22934764

[B33] Kumari R. Kumar R. C. (2014). Open source drug discovery and A. Lynn. J. Chem. Inf. Model. 54, 1951–1962. 10.1021/ci500020m 24850022

[B34] Lamb Y. N. (2020). Remdesivir: First Approval. Drugs 80, 1355–1363. 32870481 10.1007/s40265-020-01378-w PMC7459246

[B35] Lu R. Zhao X. Li J. Niu P. Yang B. Wu H.. (2020). Genomic characterization and epidemiology of 2019 novel coronavirus: implications for virus origins and receptor binding. Lancet 395, 565–574. 10.1016/S0140-6736(20)30251-8 32007145 PMC7159086

[B36] Mehta N. Mazer-Amirshahi M. Alkindi N. Pourmand A. (2020). Pharmacotherapy in COVID-19; a narrative review for emergency providers. Am. J. Emerg. Med. 38, 1488–1493. 10.1016/j.ajem.2020.04.035 32336586 PMC7158837

[B37] Mortenson P. N. Murray C. W. (2011). Assessing the lipophilicity of fragments and early hits. J. Comput. Aided Mol. Des. 25, 663–667. 10.1007/s10822-011-9435-z 21614595

[B38] Nagarajan H. Narayanaswamy S. Vetrivel U. (2020). Mutational landscape screening of methylene tetrahydrofolate reductase to predict homocystinuria associated variants: an integrative computational approach. Mutat. Res. Fundam. Mol. Mech. Mutagen. 819:111687. 10.1016/j.mrfmmm.2020.111687 31968288

[B39] Naqvi A. A. Fatima K. Mohammad T. Fatima U. Singh I. K. Singh A. (2020). Insights into SARS-CoV-2 genome, structure, evolution, pathogenesis and therapies: structural genomics approach. Biochim. Biophys. Acta Mol. Basis Dis. 13:165878 10.1016/j.bbadis.2020.165878 PMC7293463 32544429

[B40] Perlman S. (2020). Another decade, another coronavirus. N. Engl. J. Med. 382, 760–762. 10.1056/NEJMe2001126 31978944 PMC7121143

[B41] Phillips J. C. Braun R. Wang W. Gumbart J. Tajkhorshid E. Villa E.. (2005). Scalable molecular dynamics with NAMD. J. Comput. Chem. 26, 1781–1802. 10.1002/jcc.20289 16222654 PMC2486339

[B42] Roughley S. D. Hubbard R. E. (2011). How well can fragments explore accessed chemical space? A case study from heat shock protein 90: miniperspective. J. Med. Chem. 54, 3989–4005. 10.1021/jm200350g 21561141

[B43] She J. Jiang J. Ye L. Hu L. Bai C. Song Y. (2020). 2019 novel coronavirus of pneumonia in Wuhan, China: emerging attack and management strategies. Clin. Transl. Med. 9, 1–7. 10.1186/s40169-020-00271-z 32078069 PMC7033263

[B44] Su S. Wong G. Shi W. Liu J. Lai A. C. Zhou J.. (2016). Epidemiology, genetic recombination, and pathogenesis of coronaviruses. Trends Microbiol. 24, 490–502. 10.1016/j.tim.2016.03.003 27012512 PMC7125511

[B45] Subissi L. Posthuma C. C. Collet A. Zevenhoven-Dobbe J. C. Gorbalenya A. E. Decroly E.. (2014). One severe acute respiratory syndrome coronavirus protein complex integrates processive RNA polymerase and exonuclease activities. Proc. Natl. Acad. Sci. U. S. A. 111, E3900– E3909. 10.1073/pnas.1323705111 25197083 PMC4169972

[B46] Tyrrell D. A. Fielder M. (2002). Cold Wars: The Fight against the Common Cold. New York, NY: Oxford University Press.

[B47] ul Qamar M. T. Alqahtani S. M. Alamri M. A. Chen L. L. (2020). Structural basis of SARS-CoV-2 3CLpro and anti-COVID-19 drug discovery from medicinal plants. J. Pharm. Anal. 10, 313–319. 10.1016/j.jpha.2020.03.009 32296570 PMC7156227

[B48] Veber F. Johnson S. R. Cheng H. Y. Smith B. R. Ward K. W. Kopple K. D. (2002). Molecular properties that influence the oral bioavailability of drug candidate. J. Med. Chem. 45:2615–23. 10.1021/jm020017n 12036371

[B49] Wang M. Cao R. Zhang L. Yang X. Liu J. Xu M.. (2020). Remdesivir and chloroquine effectively inhibit the recently emerged novel coronavirus (2019-nCoV) *in vitro*. Cell. Res. 30, 269–271. 10.1038/s41422-020-0282-0 32020029 PMC7054408

[B50] Wrapp D. Wang N. Corbett K. S. Goldsmith J. A. Hsieh C. L. Abiona O.. (2020). Cryo-EM structure of the 2019-nCoV spike in the prefusion conformation. Science 367, 1260–1263. 10.1126/science.abb2507 32075877 PMC7164637

[B51] Wu G. Robertson D. H. Brooks C. L. III. Vieth M. (2003). Detailed analysis of grid-based molecular docking: a case study of CDOCKER—A CHARMm-based MD docking algorithm. J. Comput. Chem. 24, 1549–1562. 10.1002/jcc.10306 12925999

[B52] Yin W. Mao C. Luan X. Shen D. D. Shen Q. Su H.. (2020). Structural basis for inhibition of the RNA-dependent RNA polymerase from SARS-CoV-2 by remdesivir. Science 1:eabc1560. 10.1101/2020.04.08.032763 32358203 PMC7199908

[B53] Zaki A. M. Van Boheemen S. Bestebroer T. M. Osterhaus A. D. Fouchier R. A. (2012). Isolation of a novel coronavirus from a man with pneumonia in Saudi Arabia. N. Engl. J. Med. 367, 1814–1820. 10.1056/NEJMoa1211721 23075143

[B54] Zhu N. Zhang D. Wang W. Li X. Yang B. Song J.. (2020). A novel coronavirus from patients with pneumonia in China, 2019. N. Engl. J. Med. 382, 727–733. 10.1056/NEJMoa2001017 31978945 PMC7092803

[B55] Ziebuhr J. (2005). The coronavirus replicase, in Coronavirus Replication and Reverse Genetics. Current Topics in Microbiology and Immunology, ed L. Enjuanes (Berlin; Heidelberg: Springer), 287, 57–94. 10.1007/3-540-26765-4_3 15609509 PMC7121973

